# Whole-Transcriptome Analysis of Repeated Low-Level Sarin-Exposed Rat Hippocampus and Identification of Cerna Networks to Investigate the Mechanism of Sarin-Induced Cognitive Impairment

**DOI:** 10.3390/biology12040627

**Published:** 2023-04-20

**Authors:** Jingjing Shi, Dongxin Liu, Qian Jin, Xuejun Chen, Ruihua Zhang, Tong Shi, Siqing Zhu, Yi Zhang, Xingxing Zong, Chen Wang, Liqin Li

**Affiliations:** State Key Laboratory of NBC Protection for Civilians, Beijing 102205, China; shijingjing9287@126.com (J.S.);

**Keywords:** sarin, cognitive impairment, synaptic plasticity, whole-transcriptome, circRNA, lncRNA, miRNA, ceRNA

## Abstract

**Simple Summary:**

Sarin is a potent organophosphate nerve agent that is a representative of a chemical weapon and poses a threat to human health. In this study, the effects of repeated low-level sarin exposure on the cognitive behavior of rats were studied, and the activity of AChE activity and dendritic spine density in the hippocampus were measured. Whole-transcriptome and bioinformatics methods were used to analyze the related molecular mechanisms, and finally, the ceRNA regulatory network was constructed and analyzed. These newly discovery techniques provide a theoretical basis for the mechanism of sarin-induced cognitive impairment and also offer new insights for the research of other organophosphorus toxicants.

**Abstract:**

Sarin is a potent organophosphorus nerve agent that causes cognitive dysfunction, but its underlying molecular mechanisms are poorly understood. In this study, a rat model of repeated low-level sarin exposure was established using the subcutaneous injection of 0.4 × LD_50_ for 21 consecutive days. Sarin-exposed rats showed persistent learning and memory impairment and reduced hippocampal dendritic spine density. A whole-transcriptome analysis was applied to study the mechanism of sarin-induced cognitive impairment, and a total of 1035 differentially expressed mRNA (DEmRNA), including 44 DEmiRNA, 305 DElncRNA, and 412 DEcircRNA, were found in the hippocampus of sarin-treated rats. According to Gene Ontology (GO) annotation, Kyoto Encyclopedia of Genes and Genomes (KEGG) enrichment, and Protein–Protein Interaction (PPI) analysis, these DERNAs were mainly involved in neuronal synaptic plasticity and were related to the pathogenesis of neurodegenerative diseases. The circRNA/lncRNA–miRNA–mRNA ceRNA network was constructed, in which Circ_Fmn1, miR-741-3p, miR-764-3p, miR-871-3p, KIF1A, PTPN11, SYN1, and MT-CO3 formed one circuit, and Circ_Cacna1c, miR-10b-5p, miR-18a-5p, CACNA1C, PRKCD, and RASGRP1 constituted another circuit. The balance between the two circuits was crucial for maintaining synaptic plasticity and may be the regulatory mechanism by which sarin causes cognitive impairment. Our study reveals the ceRNA regulation mechanism of sarin exposure for the first time and provides new insights into the molecular mechanisms of other organophosphorus toxicants.

## 1. Introduction

Sarin (GB) is a powerful organophosphorus (OP) nerve agent and is a typical chemical weapon [1,2]. It is one of the main chemical warfare agents used in military equipment because of its strong toxicity and quick action. In the 1980s, the Iraqi government deployed sarin against both the Iranian army and the Iraqi Kurds [3]. During the Gulf War, an estimated 200,000 U.S. military personnel were exposed to low levels of sarin [4]. In Japan, sarin was used in a subway attack in Matsumoto and Tokyo, and about 5500 people were exposed. During the Syrian civil war, about 3600 people have been exposed [5,6]. Additionally, at military installations and in defensive areas where sarin has previously been buried, residual agents can contaminate soil and water sources, resulting in the long-term (chronic or subacute) exposure of nearby residents. These incidents indicate that sarin poses a potential threat to human life.

At present, the relevant studies on sarin mainly focus on the acute toxicity caused by high-dose exposure. The acute lethal toxic effect of sarin exposure is largely through irreversible acetylcholinesterase (AChE) inhibition, leading to the excessive stimulation of postsynaptic cholinergic receptors, and resulting in body tremors, epilepsy, salivation, muscle contractions, and ultimately, respiratory depression and death [7]. Evidence of sarin exposure suggests that long-term adverse effects can occur, even at nonconvulsive and clinically asymptomatic doses, leading to cognitive and neurobehavioral dysfunction in victims. Gulf War veterans exposed to low levels of sarin performed worse than unexposed veterans on tests of visuospatial memory and visuospatial ability [8]. During the sarin gas attack on the Tokyo subway, firefighters and medical workers who responded to the disaster also experienced a chronic decline in memory [9]. Animal study experiments have shown that a single sarin exposure causes the long-term impairment of working and reference memory function in rats, with no recovery observed during the study period [10]. Somewhat long-term memory impairment can be induced when rats are repeatedly exposed to clinically asymptomatic and nonconvulsive concentrations of sarin [11]. As observed in rhesus and African green monkeys exposed to low levels of sarin, the victims did not develop any acute cholinergic neurotoxic symptoms, but also showed cognitive decline [12]. The above results suggest that long-term exposure to repeated low levels of sarin can cause cognitive and memory impairments, but the underlying mechanisms are poorly understood. Thus, understanding the mechanism behind its action is crucial.

As we know, the hippocampus is the key brain area for cognitive functions, such as learning and memory, and some studies on organophosphates have shown that their damage to cognitive function is also caused by changes in hippocampal function. Lee et al., found that prolonged exposure to neurotoxic levels of chlorpyrifos changed the way neurotrophins and neuropeptides are expressed in the hippocampus [13]. Research by Farizatto et al., showed that paraoxon (Pxn) exposure led to alterations in early synaptic and selective adhesion signaling in the hippocampus and caused behavioral and cognitive deficits [14]. Li et al., revealed that the chronic injection of isocarbophos caused memory and learning problems, which may have been related to hippocampal malfunction [15]. In addition, several animal studies on sarin exposure and hippocampal function alteration have indicated that the hippocampus is crucial to the development of sarin-mediated cognitive impairment [16]. Studies in humans have also shown that Gulf War Veterans’ hippocampus architecture has been negatively and persistently impacted by low-level sarin exposure [17]. Despite the fact that existing studies have demonstrated that the hippocampus is one of the brain regions that is vulnerable to sarin-induced cognitive damage, the biological mechanism of this association is still unknown. Therefore, the aim of this study is to investigate the potential molecular mechanism of the impairment of hippocampal learning and memory function in rats induced by repeated low-dose sarin exposure.

Whole-transcriptome sequencing includes messenger RNA (mRNA), microRNA (miRNA), circular RNA (circRNA), long non-coding RNA (lncRNA), and other non-coding RNA sequencing [18]. Genetic information is carried by mRNA, which is produced by DNA [19]. After binding to the 3′ or 5′ UTR of a specific mRNA, miRNA regulates gene expression at the post-transcriptional level by degrading the target mRNA or inhibiting its translation [20]. LncRNAs are DNA transcript products with a length of more than 200 nucleotides that are not directly involved in the protein-coding process; instead, they regulate gene expression through cis-regulation and trans-regulation [21]. CircRNAs are endogenous non-coding RNAs with a closed loop structure, which are widely regarded as microRNA sponges and are crucial in gene expression regulation [22]. LncRNAs and circRNAs influence the subsequent transcription and translation processes by competitively binding to the metal response element of the corresponding miRNA, and comprise the competitive endogenous RNA (ceRNA) [23]. The ceRNA network, which is made up of lncRNA/circRNA–miRNA–mRNA, has received much attention and is a novel gene regulation system. Interfering with ceRNA interactions would upset the equilibrium of cellular processes and functions, which would result in a variety of diseases [24,25,26,27,28]. Transcriptomics has recently been employed by researchers to investigate the nervous system toxic effects caused by sarin. Pachiappan et al., used human nerve cells (SH-SY5Y) to explore sarin-induced neurodegenerative changes, and it is important to note that this study was conducted using cell lines, so the gene expression profiles represent single-cell type responses; this differs from findings using brain tissue to represent multiple cell types [29,30]. Spradling et al., explored the molecular mechanism of sarin-induced epilepsy by studying the transcriptional responses of neurotoxic-sensitive regions in the amygdala, cortex, and hippocampus [30]. Chan et al., studied the changes in hippocampal gene expression and pathway perturbation caused by sarin [31]. These studies have been only limited to mRNA expression changes, and the whole RNA-mediated interaction network has not been studied yet. To date, there have been no reports on the ceRNA regulatory network responsible for the cognitive impairment induced by repeated low-level sarin exposure.

## 2. Materials and Methods

### 2.1. Animals

SPF Biotechnology Co., Ltd. (Changping, Beijing, China) (license number: SCXK (Beijing) 2019-0010) provided adult male Sprague Dawley rats weighing 200 ± 5 g. The feeding environment was kept at an ambient temperature (22–25 °C), at 40–70% humidity, and under a 12–12 h light–dark cycle. A standard diet was provided, and food and water could be consumed at will. All animal experimentation was performed in strict conformity with the Guidelines for the Care and Use of Experimental Animals, and was authorized by the State Key Laboratory of NBC Protection for Civilians under permission No. LAE-2021-11-001.

### 2.2. Drugs

Sarin was provided by the Laboratory of Analytical Chemistry, Research Institute of Chemical Defense (Changping, Beijing, China), and was dissolved in isopropanol in order to prepare 10 mg/mL of storage solution. The storage liquor was diluted with 0.9% normal saline to 60 μg/mL working solution, which was prepared immediately and used up within 2 min.

### 2.3. Administration and Assignment

**Figure 1:**
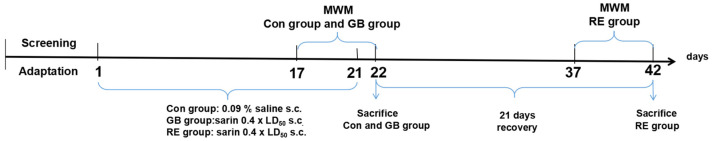
Diagram of overall study design. MWM, Morris water maze; Con, Control group; GB, sarin group; RE, recovery group.

All of the rats were maintained for a week before the experiment so that they could get used to the lab environment. In the open field test, rats with poor motor abilities were omitted, and the remaining rats were randomly assigned to three groups: the control group (Con, *n* = 15), the sarin-treated group (GB, *n* = 15), and the recovery group (RE, *n* = 15). Rats in the GB and RE groups were subcutaneously (s.c.) injected with sarin for 21 consecutive days at a dose of 40 μg/kg (0.4 × LD_50_) per day, and the same volume of 0.9% saline was given to the rats in the control group. The dose of sarin was determined based on previous studies and our unpublished data [32,33,34]. In total, 6 rats from the GB and Con groups were randomly selected for water maze training from day 17 to day 21; during this period, the sarin exposure was performed at the end of daily training. The test was performed on day 22. After the test, the rats in the Con and GB groups were anesthetized with isoflurane and rapidly decapitated on ice, and their hippocampus was separated and stored at −80 °C. The rats in the RE group were kept in the cage for 21 days for recovery, and 6 rats were selected for water maze training from day 37 to day 41. The test was performed on day 42. After the test, the rats in the RE groups were anesthetized with isoflurane and rapidly decapitated on ice, and their hippocampus was separated and stored at −80 °C. The hippocampal tissues of the rats after water maze training were selected for whole-transcriptome analysis (*n* = 6), and the remaining 9 hippocampal samples from each group were used for a subsequent AChE activity assay (*n* = 6) and Golgi staining (*n* = 3). The mental status, growth, and development of the rats were observed during the administration and recovery periods, and their body weight was recorded. The experimental procedure is shown in Figure 1. 

### 2.4. Morris Water Maze Test

The rats’ learning and memory capacities were measured using the Morris water maze test to evaluate the degree of cognitive impairment in each group. The procedure was partially changed according to the literature [35]. The experiment included positioning navigation training and the space exploration test. 

For positioning navigation training, the pool was divided into four quadrants, with the platform placed in the center of one of the quadrants. Rats were placed with their head toward the wall of the pool in the water, and the time was recorded until the rat found the hidden underwater platform. If this period of time was more than 60 s, the rats were led to a platform and kept there for 10 s. Each rat trained continuously for 5 days, 4 times a day, with a 30 min interval between training sessions.

For the space exploration test, the platform was removed and the rat was placed in water for 60 s probe trial. As measures of spatial memory, the amount of time spent in the target quadrant (the quadrant where the platform was placed) and the number of entrances into the quadrant were noted.

### 2.5. AChE Activity Assay

To evaluate the sarin-induced toxicity, AChE activity in the hippocampus was analyzed. For that, 0.02–1.0 g of frozen hippocampus tissue was homogenized with PBS (0.01 M, pH 7.4) at 2–8 °C. The precooled lysis buffer was added to the homogenate at a weight (g) to volume (mL) ratio of 1:9 and centrifuged at 8000× *g* for 10 min. The specified procedure was carried out in accordance with the manufacturer’s instructions, using the supernatant for the enzyme activity assay (Elabscience, Wuhan, China).

### 2.6. Golgi Staining and Counting

The procedure was carried out in accordance with the instructions of the FD Rapid Golgi Stain Kit (FD Neuro Technologies, Columbia, MD, USA). The hippocampus tissues of rats were collected and immersed in a mixture of solution A and solution B (prepared 24 h in advance). The mixture was replaced the next day, and kept at room temperature for two weeks in the dark. The brain tissue was taken out, immersed in solution C, and kept in the dark for 24 h. The solution was then replaced for another 4 days. The tissues were taken and cooled into blocks in precooled isopentane, and the surface isopentane was wiped off. The tissue was cut into approximately 100 μm sections using a cryostat microtome (Leica, Wetzlar, Hessen, Germany) and attached to slides. Then, the slides were placed in the mixture of solution D and solution E. After the reaction, the slides were washed with distilled water and dehydrated in gradient ethanol for 60 s each time. The tissues were cleared with a xylene solution for 2 min. Finally, neutral resin was used to seal the discs, and the images were observed under a microscope (Nikon Corporation, Minato, Tokyo, Japan). The density and length of the dendritic spines were analyzed using Image-Pro Plus 6.0 software (Media Cybernetics, Silver Spring, MD, USA). 

### 2.7. RNA Isolation, Library Construction, and Sequencing 

RNA isolation, library construction, and sequencing were performed at Shanghai Majorbio Bio-pharm Biotechnology Co., Ltd. (Minhang, Shanghai, China). See the manufacturer’s website (https://cloud.majorbio.com/ (accessed on 5 March 2022)) and its online instructions for the specific experimental procedures.

### 2.8. Gene Expression Level and Differential Expression Analysis

To gather information about the miRNA and ncRNA annotations, the reads that matched the reference genome were conformed with the miRBase (http://www.mirbase.org/ (accessed on 19 July 2022)) and Rfam (https://rfam.org/search (accessed on 19 July 2022)) databases, respectively. In order to further analyze the DEmRNA, DElncRNA, and DEcircRNA among the different samples and reveal the regulatory mechanism of the genes by combining sequence functional information, the software RSEM (http://deweylab.biostat.wisc.edu/rsem/ (accessed on 19 July 2022)) was applied to quantitatively analyze the overall expression levels of mRNA, lncRNA, and circRNA at the gene level or transcript level. The regulatory mechanism of the genes was discovered by combining the sequencing and function information, and the expression level was homogenized using the reads per million mapped reads (RPM) method. In essence, all of the aforementioned differential expression analyses were carried out using the DESeq2 package, and RNAs with |log2FC| > 1.5 and *p*-value < 0.05 were regarded as significant DERNAs.

### 2.9. Gene Ontology Annotations, Kyoto Encyclopedia of Genes and Genomes Pathway Analysis, and Protein–Protein Interaction Network of DEGs

The DERNAs and anticipated target genes were annotated using the Gene ontology annotations (GO) and Kyoto Encyclopedia of Genes and Genomes (KEGG) databases. The top 20 highly enriched GO and KEGG pathways were utilized to build the interaction network of the signaling pathways of the DERNAs and their targets based on the enrichment score, at a *p*-value < 0.05, compared to the background of the entire genes. Goatools (https://github.com/tanghaibao/Goatools (accessed on 16 August 2022)) and KOBAS (http://kobas.cbi.pku.edu.cn/home.do (accessed on 16 August 2022)) were used to perform GO annotations and KEGG pathway analysis. The DERNAs were added into the STRING database (https://string-db.org/ (accessed on 22 August 2022)) to build a protein–protein interaction (PPI) network. Visual presentation and network optimization were performed using Cytoscape software (https://cytoscape.org/ (accessed on 22 August 2022)). Molecular Complex Detection (MCODE, https://apps.cytoscape.org/apps/MCODE (accessed on 22 August 2022)) was used to perform the PPI network module analysis to provide meaningful protein modules and cluster scores.

### 2.10. ceRNAs Regulatory Network Analysis

Based on the sequences of the miRNA response elements, the Targetscan (http://www.targetscan.org/ (accessed on 5 September 2022)) and Miranda (http://www.miranda.org/ (accessed on 5 September 2022)) programs were applied to estimate the binding sites of target RNAs and miRNAs. The CircRNA/lncRNA–miRNA–mRNA regulation networks were used to illuminate the functions and relationships among the DEncRNAs and DEmRNAs, and the relationships among them were created and depicted using the Cytoscape program.

### 2.11. Statistical Analyses

GraphPad Prism 8 (GraphPad Software Inc., La Jolla, CA, USA) and SPSS 26.0 (IBM Corp., Armonk, NY, USA) were applied to conduct statistical analysis; all data are shown as the mean ± SD. The Shapiro–Wilk test was used to assess the normal distribution of the data. If the variables conformed to normal distribution, one-way analysis of variance (ANOVA), followed by the Bonferroni test, was used. If variables did not conform to a normal distribution, the nonparametric Kruskal–Wallis test, followed by Dunn’s test, was performed. For all analyses, * *p*-value < 0.05 was considered statistically significant.

## 3. Results

### 3.1. Sarin Irreversibly Impaired the Learning and Memory Functions of Rats

**Figure 2:**
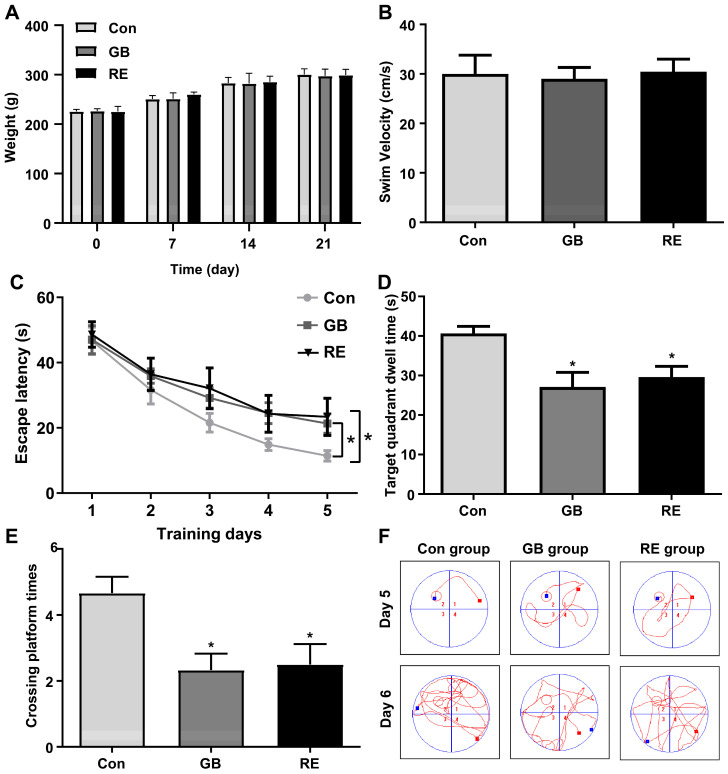
Morris water maze results of rats in different groups. (**A**) Changes in body weight of rats during drug administration. (**B**) The average swimming speed of the rats. (**C**) The escape latency in the positioning navigation test. (**D**) The crossing platform times in the space exploration test. (**E**) The target quadrant dwell time in the space exploration test. (**F**) Representative swimming trajectories of each group on days 5 and 6. The red squares represent drop points and green squares represent exit points. Data are presented as mean ± SD. *n* = 6, * *p* < 0.05, compared with Con. Data of escape latency were analyzed using non-parametric Kruskal–Wallis test followed by Dunn’s test, and other data were analyzed using one-way ANOVA followed by the Bonferroni test.

A schematic representation of the rat models used for repeated low-level sarin exposure and prolonged recovery after exposure is shown in Figure 1. During the experiment period, the rats in each group did not display any diet and water consumption abnormalities. There was no muscle tremor, convulsion, or death. After statistical analysis, the weight growth rate of the rats in the sarin-treated group (GB) and recovery group (RE) in the first three weeks were close to that of the saline group (Con), and there was no significant difference in the body weight and average swimming speed of the rats in each group (Figure 2A,B), thereby excluding any potential interference caused by the rats’ different physical abilities. In the water maze test, the spatial learning and memory capacities of the rats were measured by escape latency, and the memory retention capacity of the rats compared to the original platform placement was measured by the number of platform crossings and the target quadrant’s search time. The results of the positioning navigation test showed that the escape latency of the rats in each group decreased gradually with the increase in the training time. As shown in Figure 2C, the escape latency of the rats in the GB group and RE group on the 3rd, 4th, and 5th day was longer than that of the Con group, and that the difference was significant on the 5th day (*p* = 0.045), demonstrating that the learning ability of the rats exposed to sarin decreased. The results of the space exploration test on the 6th day are shown in Figure 2D,E. The rats in the GB and RE groups crossed the platform and stayed in the target quadrant significantly less than those in the con group, and this difference was statistically significant (F_Crossing platform times_ = 5.83, *p* = 0.013, and F_Target quadrant dwell time_ = 6.43, *p* = 0.0096), while there was no statistically significant difference between the GB group and the RE group. Analysis of the swimming trajectories of the rats in each group on the 5th and 6th days showed that compared with the con group, the swimming trajectories of the rats in the GB and RE groups were significantly disordered and purposeless (Figure 2F). These results suggest that repeated low-level sarin exposure impairs spatial learning and memory in rats, with no improvement observed after prolonged recovery.

### 3.2. Effects of Sarin on AChE Activity and Dendritic Spines Density in Rat Hippocampus

**Figure 3:**
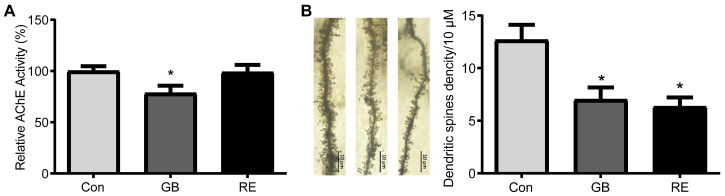
Effects of sarin on AChE activity and dendritic spine density in the rat hippocampus. (**A**) AChE activity in rat hippocampus. Data are presented as mean ± SD. *n* = 6, * *p* < 0.05, one-way ANOVA followed by Bonferroni test, compared with Con. (**B**) Density of dendritic spines in hippocampal neurons. Data are presented as mean ± SD. *n* = 3, * *p* < 0.05, one-way ANOVA followed by Bonferroni test, compared with Con. The left represents a dendritic spine structure at 100× magnification, and the right represents the results of the dendritic spine density analysis.

The hippocampus tissue of the rats was homogenized and the AChE activity was detected. AChE activity in the hippocampus decreased significantly in the rats of the GB group (Figure 3A) compared to the control group (F _(2, 15)_ = 3.75, *p* = 0.027), by nearly 20%, while the rats in the RE group exhibited no difference in AChE activity after 21 days of recovery. This suggests that repeated low-level sarin exposure induces changes in the AChE activity in the hippocampus of rats that are not persistent, but can return to normal levels over time. The Golgi staining results and the quantitative statistics of the dendritic spine structures in the rat hippocampus are shown in Figure 3B. The findings demonstrate that the dendritic spine density of the hippocampal neurons in the GB and RE groups was significantly lower than that of the Con group (F _(2, 6)_ = 8.12, *p* = 0.019). Moreover, no differences between the GB and RE groups were discovered. This shows that a long-term reduction in the dendritic spine density in the rat hippocampus neurons following repeated low-level sarin exposure is independent of AChE activity. The above results further suggest that repeated low-dose sarin exposure causes persistent changes in the synaptic structure, most likely through non-cholinergic pathways rather than changes in AChE activity.

### 3.3. Differential Expression Analysis

**Figure 4:**
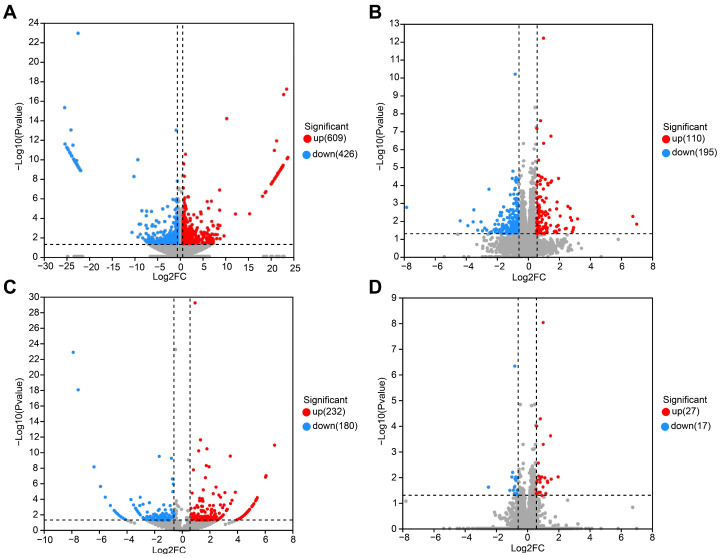
Differential expression of mRNA, lncRNA, circRNA, and miRNA in hippocampus after sarin exposure. (**A**) Volcano plot of differentially expressed mRNA. (**B**) Volcano plot of differentially expressed lncRNA. (**C**) Volcano plot of differentially expressed circRNA. (**D**) Volcano plot of differentially expressed miRNA. The red dots represent the up-regulated transcripts, while the blue dots represent the down-regulated transcripts, compared with the control group.

**Figure 5:**
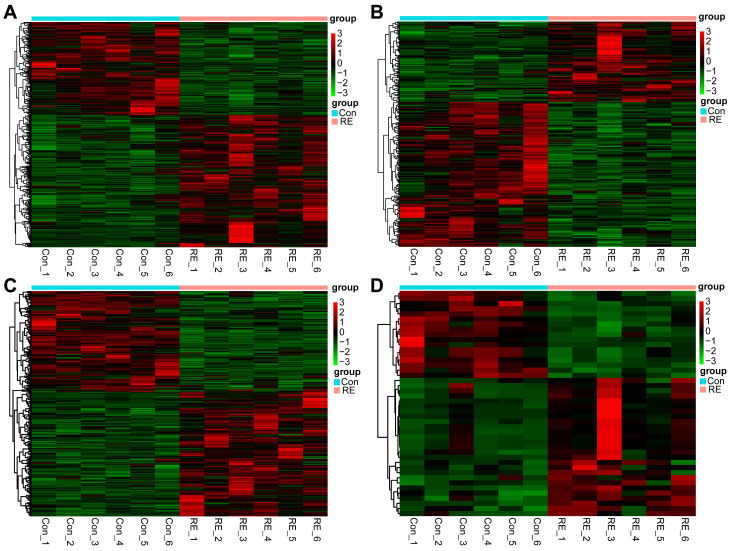
Hierarchical clustering and heat map analysis of DERNAs. (**A)** DEmRNA, (**B**) DElncRNA, (**C**) DEcircRNA, and (**D**) DEmiRNA in each group. Red represents the up-regulated RNAs, while green represents the down-regulated RNAs, compared with the control group.

In order to study the non-cholinergic mechanism of the long-term memory impairment that is induced by sarin exposure, whole-transcriptome sequencing was carried out on the hippocampus of rats in the saline control group (Con) and the group that underwent a 21-day recovery after sarin exposure (RE). The hippocampal samples of the two groups of rats (*n* = 6) that completed the behavioral experiments were taken out, and 12 samples were then sequenced and analyzed. According to a prespecified threshold (|log2FC| > 1.5, *p*-value < 0.05), a total of 1035 DEmRNAs, including 609 up-regulated and 426 down-regulated (Figure 4A), 305 DElncRNA, including 110 up-regulated and 195 down-regulated (Figure 4B), 412 DEcicRNA, including 232 up-regulated and 180 down-regulated (Figure 4C), and 44 DEmiRNA, including 27 up-regulated and 17 down-regulated (Figure 4D), were obtained in the RE group compared to the Con group. Hiplot (https://hiplot.com.cn/ (accessed on 13 October 2022)) was used to draw heat maps and visualize these DERNAs. The clustering heat maps of DEmRNA, DEmiRNA, DElncRNA, and DEcircRNA are shown in Figure 5A–D. We discovered that the sarin-exposed samples were significantly separated from the saline control samples, indicating that the results of the differential expression analysis were trustworthy. 

### 3.4. GO Annotation and KEGG Pathway Analysis

**Figure 6:**
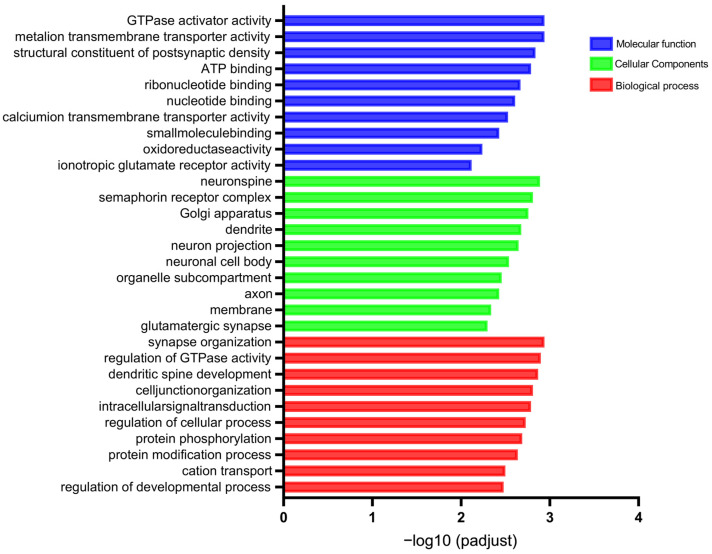
Top 20 GO term annotation analysis. Red bars represent biological processes, green bars represent cellular composition, and blue bars represent molecular functions.

**Figure 7:**
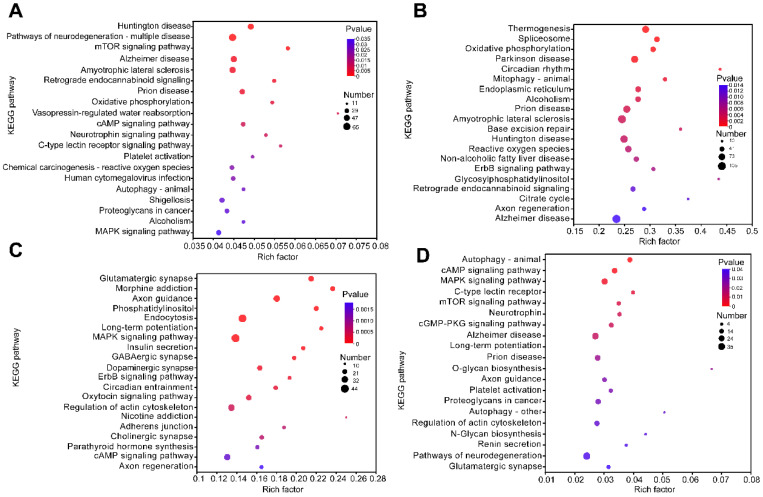
KEGG pathway enrichment analysis. (**A**) Top 20 pathways enriched by DEmRNAs. (**B**) Top 20 pathways enriched by target genes of DElncRNAs. (**C**) Top 20 pathways enriched by target genes of DEcircRNAs. (**D**) Top 20 pathways enriched by target genes of DEmiRNAs. The size of the dots represents the number of enriched genes in the pathway. The color of the origin represents the significance level of the enriched pathway, which increases gradually from the blue to red color.

To investigate the role that DERNAs play in the deficiency in memory and learning that is aroused by sarin exposure, GO annotation, in order to analyze the functions of DEmRNAs in the saline control group and that group that underwent a 21-day recovery after sarin exposure, was performed. As shown in Figure 6, the highly enriched GO terms in the categories of Molecular Function (MF), Cellular Components (CC), and Biological Process (BP) were represented as bar graphs. These terms included GTPase activator activity, ion transmembrane transporter activity, structural constituent of postsynaptic density, neuron spine, dendrite, neuron projection, synapse organization, regulation of GTPase activity, dendritic spine development, etc. These are essentially clustered in synaptic function. In order to confirm the signaling cascades linked to the DEmRNAs, KEGG pathway analysis was also carried out, and the results were displayed as bubble plots. The top 20 significantly enriched pathways are shown in Figure 7A, including the mTOR signaling pathway, the Huntington disease pathway, retrograde endocannabinoid signaling, the pathways of neurodegeneration–multiple diseases, that of prion disease, amyotrophic lateral sclerosis, oxidative phosphorylation, Alzheimer’s disease, and so on. These pathways frequently result in cognitive impairment and have been linked to the emergence of neurodegenerative disorders. Meanwhile, an enrichment analysis of the DEmiRNA, DEcicRNA, and DElncRNA-related target genes was performed, and the results are shown in Figure 7B–D. The top enriched pathways were mainly associated with mediating signal transduction and nervous system diseases. These included, for example, the cAMP signaling pathway, MAPK signaling pathway, morphine addiction, axon guidance, oxidative phosphorylation, Parkinson’s disease, and the glutamatergic synapse pathway. Combining the above GO and KEGG analysis results, it is most likely that the learning and memory impairment induced by repeated low-level sarin exposure is related to changes in the expression levels of synapse-related genes, which are involved in cognitively associated neurodegenerative diseases.

### 3.5. PPI Network Analysis

**Figure 8:**
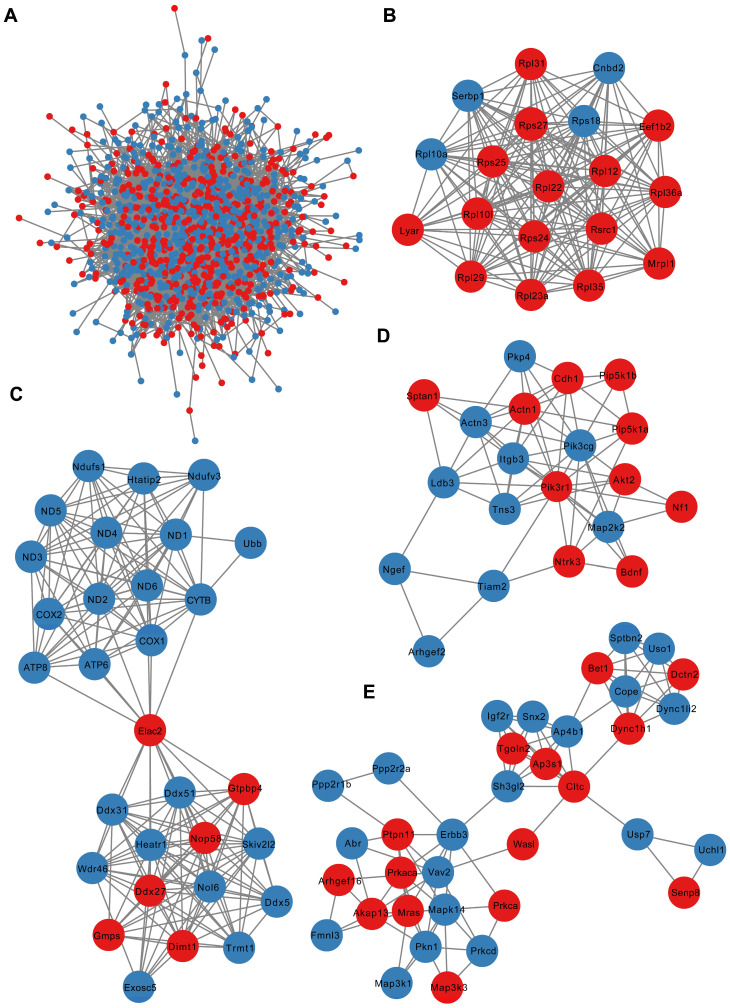
PPI network of DEmRNAs. (**A**) PPI network of all DEmRNAs. (**B**–**E**) Significantly clustered four modules from the PPI network. Red nodes represent up-regulated DEmRNAs, blue nodes indicate down-regulated DEmRNAs and the lines between two points indicate protein–protein interactions.

As shown in Figure 8A, the PPI network of all the DEmRNAs in the saline control group and the 21-day recovery after sarin exposure group was built using the String database to detect the connections between these DEmRNAs. The PPI network was visualized using the Cytoscape program, and the MCODE plug-in of Cytoscape was used to build a sub-network to identify the hub modules and important genes in the PPI network. Here, we highlighted the four modules with the closest interactions. Module 1 (score = 17.8, Figure 8B) contained 19 nodes and 218 interaction pairs, and the related genes were mainly engaged in genetic information processing and the translation pathway. Module 2 (score = 12.8, Figure 8C) contained 30 nodes and 306 interaction pairs, and the related genes were mainly enriched in oxidative phosphorylation, Alzheimer’s disease, Huntington’s disease, Parkinson’s disease, and neurodegeneration–multiple disease pathways. Module 3 (score = 7.2, Figure 8D) contained 20 nodes and 68 interaction pairs, and the genes were mainly related to phospholipase D, neurotrophin, and the PI3K-Akt signaling pathway. Module 4 (score = 5.5, Figure 8E) contained 35 nodes and 91 interaction pairs, and the genes were primarily abundant in the GnRH and MAPK signaling pathways. These genes in the above four modules are closely related to synaptic plasticity, which further suggests that the impairment of learning and memory caused by repeated low-level sarin exposure may be related to the dysregulation of gene expression related to nervous system diseases.

### 3.6. Competitive Endogenous RNA Network of Different Expression of mRNAs, lncRNAs, circRNAs, and miRNAs 

**Figure 9:**
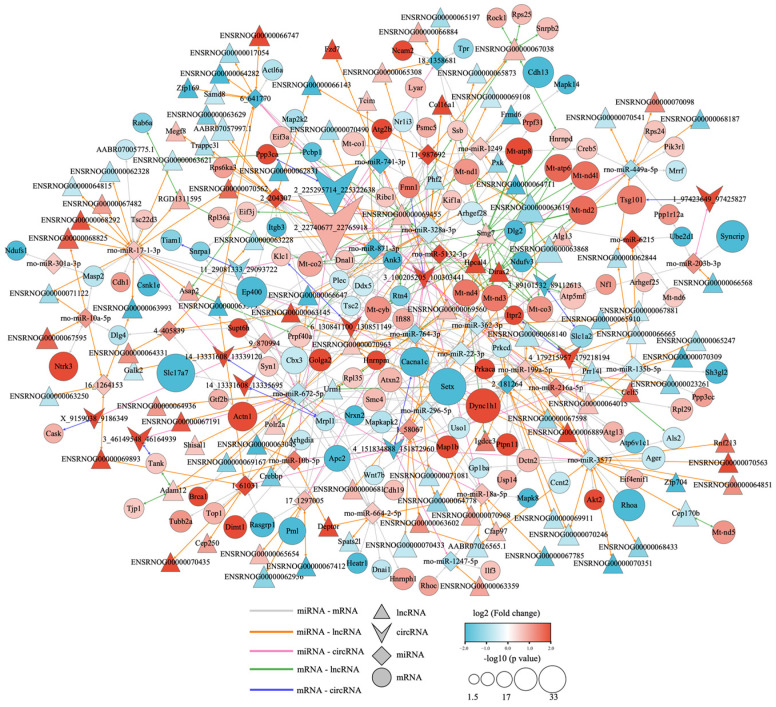
The lncRNA/circRNA–miRNA–mRNA network. The red color indicates up-regulated RNAs, while blue indicates down-regulated RNAs. mRNAs are represented by circular nodes, miRNAs are represented by prism nodes, lncRNAs are represented by triangle nodes, and circRNAs are represented by arrow nodes.

**Figure 10:**
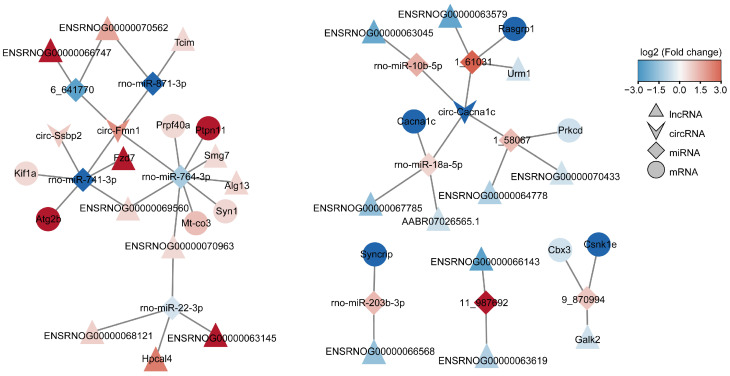
The ceRNA network with up- and down-regulated relationships. Red color indicates up-regulated RNAs, while blue indicates down-regulated RNAs. mRNAs are represented by circular nodes, miRNAs are represented by prism nodes, lncRNAs are represented by triangle nodes, and circRNAs are represented by arrow nodes.

The target mRNAs of the miRNAs were predicted using the miRanda database, and the target miRNAs of the lncRNAs and circRNAs were predicted using the starBase database. The network of the connections between lncRNA–miRNA–mRNA and circRNA–miRNA–mRNA was built based on the regulatory relationships between DEmiRNA-DEmRNA, DEmiRNA–DElncRNA, DEmiRNA–DEcircRNA, DEmRNA–DElncRNA, and DEmRNA–DEcircRNA. As shown in Figure 9, a total of 264 lncRNA–miRNA–mRNA and 109 circRNA–miRNA–mRNA interactions were eventually discovered, of which 49 lncRNAs, 5 circRNAs, 16 miRNAs, and 78 mRNAs were down-regulated, along with 38 lncRNAs, 9 circRNAs, 20 miRNAs, and 116 mRNAs that were up-regulated. To make the regulatory relationship clearer, on this basis, according to the strict up–down regulatory relationship between RNAs and the co-expression relationship between DEmRNA–DElncRNA and DEmRNA–DEcircRNA, we constructed the ceRNA network on the basis of the interactions mentioned above in order to further clarify the regulatory relationship. As shown in Figure 10, in two cases with expression regulatory linkages, lncRNA/circRNA (down-regulated)–miRNA (up-regulated)–mRNA (down-regulated) or lncRNA/circRNA (up-regulated)–miRNA (down-regulated)–mRNA (up-regulated), were covered by the tight ceRNA network. Among the known genes, miR-741-3p, miR-764-3p, miR-871-3p, and miR-22-3p were down-regulated and their target mRNAs, KIF1A, ATG2B, PRPF40A, PTPN11, SYN1, and MT-CO3, were up-regulated. Multiple lncRNAs/circRNAs, including Lnc_Tcim, Lnc_Hpcal4, Lnc_Fzd7, Lnc_Smg7, Lnc_Alg13, Circ_Ssbp2, and Circ_Fmn1, act as miRNA sponges to control the expression of these miRNA target mRNAs. In addition, the expressions of miR-10b-5p, miR-18a-5p, and miR-203b-3p were up-regulated, and their target genes, RASGRA1, CACNA1C, SYNCRIP, CBX3, and CSNK1Ec, were down-regulated. The expressions of Lnc_Urm1, Lnc_Galk2 and Circ_Cacna1c, which regulated these miRNAs, were down-regulated. These RNAs and their interactions may provide new insights into the mechanisms of repeated low-level sarin-induced learning and memory impairment. 

## 4. Discussion

Previous studies on the effects of sarin on animal behavior have mostly focused on the sublethal dose, convulsive dose, and the acute effect of the sub-convulsive dose. However, little is known about the neurotoxicology of repeated low-level exposure to sarin. Studies on sarin have suggested that the maximum tolerated dose (MTD) of sarin that could be administered for 2 to 4 weeks of exposure without evidence of cholinergic toxicity is 0.4 × LD_50_ [36]. There were no differences in the weight gain, body temperature, complete blood cell count, blood chemistry, and histopathology between animals at this dose and in vehicle controls. Therefore, the dose of 0.4 × LD_50_ has been used in a variety of chronic or subacute exposure models [32,33,34,37,38]. In this study, a rat model of repeated low-level sarin exposure was established by using a subcutaneous injection of 0.4 × LD_50_ (40 μg/kg) for 21 consecutive days. Rats exposed to sarin experienced learning and memory impairment that persisted for at least 3 weeks after the exposure ended. The hippocampus is a crucial brain region that mediates learning and memory, but the AChE activity in the hippocampus of the cognitively unfunctional rats in this study was not persistently inhibited, and the sarin-exposed rats and the saline control rats showed no differences in their body weight, diet, or exercise. Meanwhile, there were no obvious cholinergic symptoms caused by organophosphate poisoning, such as diarrhea, sweating, convulsion, or death. These results suggest a possible dissociation between repeated low-level sarin-induced neurotoxicity and its well-known cholinergic effects, thus revealing additional (non-cholinergic) mechanisms that mediate the cognitive damage caused by sarin.

Long-term exposure to OPs can result in cognitive damage, including learning and memory problems. The Morris water maze is a method that is frequently used in the study of neuroscience to assess rodents’ capacity for learning and memory [35]. In this study, rats treated with 40 μg/kg sarin, either without or with recovery, had prolonged escape latency, shortened target quadrant residence time, and reduced platform crossing times. These findings were consistent with prior reports on the cognitive impairment induced by repeated low-level sarin exposure and demonstrated that repeated low-level sarin exposure could produce the long-term impairment of learning and memory function in rats [11]. Cognitive functions are affected by a variety of regulatory mechanisms in the brain. For a period of time, the inhibition of AChE activity was thought to be the main mechanism by which OPs caused neurotoxicity, including cognitive impairment. In the study of OPs such as sarin, soman and dichlorvos, it has been found that OP poisoning can significantly inhibit the activity of AChE, resulting in the accumulation of ACh in the synaptic cleft, causing a series of neurotoxic effects [2,39,40]. Abou-Donia et al., suggested that the sarin-sustained inhibition of AChE can cause the excessive activation of ACh in the hippocampus, and that the excessive accumulation of ACh leads to cholinergic crisis, which is ultimately responsible for hippocampal damage, leading to cognitive defects. However, some evidence has shown that cognitive impairment persists long after repeated low-level exposure to OPs, although AChE activity is restored to normal, so this persistent impairment may be independent of AChE inhibition. For example, Moser et al., found that continuous exposure of 1 mg/kg of chlorpyrifos for 1 year had no significant effect on AChE activity in the brains of rats [41], but that the rats exposed to chlorpyrifos experienced spatial memory impairment. Some studies on low-dose sarin exposure have shown that the AChE activity in the cortex and blood decreased within 24 to 48 h after 0.4 × LD_50_ sarin exposure, but returned to normal within 48 to 600 h after exposure [33]. Scremin et al., found that after 3 weeks of the continuous exposure of rats to 0.5 × LD_50_ sarin, there was no change in the AChE activity of the hippocampus from week 2 to week 26 after the last exposure [42]. In the present study, rats exposed to 0.4 × LD_50_ sarin for 3 weeks showed a decrease in AChE activity in the hippocampus 24 h after the last exposure. However, cognitive impairment persisted even after the AChE activity was restored to control levels 21 days after the last exposure. Although sarin-induced changes in AChE were similar to those documented in the literature, AChE inhibition alone was unable to account for the prolonged cognitive impairment.

Synaptic plasticity in the hippocampus is well recognized as being directly tied to learning and memory, and dendritic spines have been considered as the morphological foundation of synaptic plasticity. Studies have shown that a reduction in the dendritic spine density leads to the weakening of synaptic plasticity, which in turn affects learning and memory function [43,44]. According to research, the damage to synaptic plasticity caused by OPs is not only impacted by ACh and other neurotransmitters, but is also linked to the activation of the learning and memory-related signaling systems, the degradation of neuronal cytoskeleton, and neurogenesis [45]. Howard et al., found OP-induced morphological alterations in axons and dendrites in neuronal cell lines [46]. Roy et al., discovered that neonatal and adult rats exposed to low levels of chlorpyrifos experienced neuronal death in the hippocampus, resulting in cognitive and behavioral impairments [47]. Campana et al., revealed that the morphology of dendrites in the prefrontal cortex (PFC) and hippocampal neurons of mice exposed to low-dose malathion was significantly changed, and the cognitive behavior of the mice was also affected [48]. These studies suggest that low-dose OP exposure may impair synaptic morphogenesis in the brain, which in turn impairs neuronal connectivity, cognition, and behavior. At present, there is no research that describes sarin exposure that leads to changes in the hippocampal dendritic spines and subsequent cognitive impairment. Our study reveals for the first time that repeated low-level sarin exposure could lead to changes in synaptic structure and cause learning and memory impairment. In this study, we further elucidate the molecular basis and regulatory mechanisms.

Finding a new toxicity mechanism is like searching for a needle in a haystack, but RNA transcriptomics technology can provide valuable clues for researchers. The use of RNA-sequencing technology, a high-throughput screening approach that can identify the expression of coding and non-coding RNAs in tissues or cells, has significant meaning for research on RNA biology from a variety of angles, including structure, gene expression, and translation. Transcriptomics has become increasingly used in toxicological investigation in recent years, and numerous DEGs and ncRNAs have been discovered. In this study, the hippocampus of rats in the saline control group and the group exposed to sarin undergoing 21 days of recovery underwent whole-transcriptome analysis. This is the first time that whole-transcriptome analysis has been used to analyze sarin, and it offers a new starting point for the investigation of the neurotoxic effects of sarin. Using whole-transcriptome study, several DEmRNAs, DEmiRNAs, DElncRNAs, and DEcircRNAs were identified in sarin-exposed rats. The GO enrichment and KEGG pathway analyses were carried out to comprehend the role of DERNAs in the primary signal transduction pathways in which they are involved. By GO terms, we found the significant enrichment of the regulation of vesicle-mediated transport, cell communication, and postsynaptic neurotransmitter receptor activity, which is the fundamental mechanism of dynamic synaptic strength modulation and is essential for the creation and regulation of synaptic function [49]. The KEGG pathway results revealed that the differentially expressed RNA or target molecules were mainly concentrated in the m-TOR, cAMP and MAPK signaling pathways, oxidative phosphorylation, axon guidance of glutamatergic synapses, and so on. Alterations in these signaling pathways are associated with nervous system-related diseases such as Huntington’s disease, Parkinson’s disease, and Alzheimer’s disease. The pathogenesis of these neurodegenerative diseases was also considered to be related to OP exposure [50]. For example, the mTOR pathway is a major regulator of synaptic plasticity and memory in the hippocampus, is involved in spatial memory and object recognition, and its modulation may help to treat cognitive dysfunction [51,52,53]. cAMP signaling in the brain mediates many neural processes, such as development, synaptic plasticity, cognition, neuropathy, and drug abuse [54]. Oxidative phosphorylation, a metabolic pathway through which mitochondria produce ATP, results in the significant impairment of LTP, and induces neuronal apoptosis and neurodegeneration [55]. Glutamatergic synapses are the main excitatory synapses in the hippocampus, which are also the structural bases of synaptic plasticity and memory formation [56]. We also constructed a PPI network to further define the functional relationships between DEmRNAs and uncover the hub genes. The findings showed that DEmRNAs abundant in the four modules with higher scores were mainly involved in ribosomal proteins, mitochondrially encoded proteins, phosphatidylinositol kinases, and mitogen-activated protein kinases [57]. Ribosomal proteins are associated with the synthesis of synaptic proteins and are essential for embryonic nervous system and brain development. Mitochondrially encoded proteins are related to mitochondrial functional integrity, cytochrome oxidase activity, and mitochondrial ATP, which are involved in the impairment of hippocampal-mediated learning and memory, motor learning, and coordination in AD mice [58]. Phosphatidylinositol kinase is mainly reported in the PI3K-Akt-mTOR signaling pathway, which has been linked to synaptic plasticity changes induced by aluminum exposure [59]. Genes related to mitogen-activated protein kinases have been related to neuronal synaptic plasticity and the regulation of long-term changes in synaptic efficacy, including long-term depression (LTD) and LTP [60]. These findings suggest that altered synaptic plasticity in the hippocampus caused by DERNAs may be a potential mechanism involved in the cognitive dysfunction induced by repeated low-level sarin exposure.

The three most significant non-coding RNA families—lncRNA, circRNA, and miRNA—have been confirmed to play various roles in the regulation of transcription and are currently a hot topic in the study of neurotoxicology [61,62,63,64,65,66,67]. According to the mechanism of ceRNA, LncRNA and CircRNA can act as a miRNA sponge, reduce the activity of miRNA by binding to it, and hinder the inhibition of miRNA on target genes, all of which contribute to the occurrence and progression of diseases. Here, we constructed circRNA/lncRNA–miRNA–mRNA ceRNA networks based on DERNAs in sarin-exposed rats. According to the regulatory relationship between these RNAs, strict restriction requirements were used to screen out the circRNA or lncRNA associated-ceRNA networks that were most likely involved in cognitive impairment. In total, 9 miRNA, 9 mRNA, 17 lncRNA, and 3 circRNA were finally screened out. Among them, up-regulated circ_Fmn1 and down-regulated circ_cacna1c were at the core of the regulatory network, negatively regulating the levels of miR-741-3p, miR-764-3p, miR-871-3p, miR-10b-5p, and miR-18a-5p. These miRNAs can further regulate their downstream target mRNAs. There is little research on circ_Fmn1, but the available information indicates that it modulates the morphology and synaptogenesis of hippocampal neurons, altering actin polymerization, spine density, dendritic development, and the long-term forms of synaptic plasticity, such as LTD and LTP [68]. Circ_Cacna1c was involved in the ERK signaling pathway and altered cognitive functions and synaptic plasticity [69]. Studies have shown that these miRNAs may be associated with neurological diseases. For example, miR-764-3p was down-regulated in the hippocampus of rats with dementia [70]. miR-741-3p was upregulated in attention-deficit/hyperactivity disorder rats [71]. miR-18a-5p was involved in axonogenesis in rat hippocampus by regulating its target genes [72]. miR-10b-5p suppressed the calcium signaling pathway and LTP. The up-regulation of miR-10b-5p in the hippocampus reduced the BDNF level and caused cognition impairment in mice [73,74]. In addition, miR-10b-5p has been observed to facilitate the differentiation of striatal neurons and be associated with Huntington’s disease [75]. The differentially expressed lncRNAs, such as Lnc_Tcim, Lnc_Hpcal4, Lnc_Fzd7, Lnc_Smg7, and Lnc_aAtg13, had binding sites with the above miRNAs and were involved in the regulation of cognition by regulating the corresponding miRNAs. For example, Lnc_Smg7-mediated RNA degradation has been implicated in neurodegenerative diseases. Lnc Hpcal4 may serve as a prognostic indicator of cognitive deterioration in Alzheimer’s patients [76]. Lnc_Fzd7 increases synaptic AMPAR localization and participates in LTP-mediated spine plasticity via CaMKII, PKA, and ERK cascades [77]. Lnc_Atg13 was an important component of the mTOR pathway, which contributes to the synthesis of synaptic proteins and is strongly linked to the pathophysiology of Alzheimer’s disease [78]. Additionally, through the literature review, it was found that the target genes of miRNA, such as KIF1A, PTPN11, SYN1, and MT-CO3, are directly linked to the incidence of neurological illnesses by regulating synaptic plasticity. KIF1A, for instance, is a key anterograde motor protein that is essential to the axonal transport of dense core vesicles in hippocampus neurons, which was also implicated in learning and memory [79]. PTPN11 is a major regulator in the Ras-MAPK pathway and mutations of the PTPN11 gene can affect pathway function, leading to cognitive-behavioral deficits [80]. SYN1, which is found in abundance in the synaptic vesicles and is connected to synapse formation, is crucial in maintaining synaptic plasticity and memory consolidation [81]. MT-CO3 is involved in mitochondrial energy metabolism and is intimately associated with the pathogenesis of Alzheimer’s disease. Knockdown of hippocampal CACNA1C resulted in a significant reduction in ERK and CREB activation in the hippocampus and reduced synaptic plasticity in glutamatergic neurons [82]. In addition, PRKCD, RASGRP1, SYNCRIP, CSNK1E, and CBX3 were involved in the regulation of synaptic plasticity and were associated with cognitive impairment caused by neurological diseases [83,84,85,86,87]. The above information and results suggest that changes in the expression levels of these RNAs are associated with alterations in synaptic function. At the same time, these RNAs were all enriched in our ceRNA network and formed two lines between them. On the one hand, increased Circ Fmn1 in the above ceRNA network resulted in the decreased expression of miR-741-3p, miR-764-3p, and miR-871-3p, and the increased expression of the target corresponding mRNAs KIF1A, PTPN11, SYN1, and MT-CO3. On the other hand, the down-regulation of Circ_Cacna1c resulted in the up-regulation of miR-10b-5p and miR-18a-5p, which in turn led to the down-regulation of the regulated target mRNAs CACNA1C, PRKCD, and RASGRP1. The balance between these two pathways is the key to maintaining the stability of synaptic function, and a disruption in this balance eventually leads to the damage of synaptic function and causes cognitive impairment, which is most likely the possible mechanism of repeated low-level sarin-induced learning and memory impairment. Notwithstanding these findings, there are certain limitations to our study. In terms of tissue selection, we only chose the hippocampus for study, which is most related to learning and memory, but the brain is a complex network, and various brain regions may affect each other. Therefore, future research needs to select other parts of the brain, such as the cerebral cortex and striatum, for deep sequencing analysis to better understand the brain neural circuitry of sarin affecting cognitive function. On the other hand, this study only focused on the related RNAs of sarin exposure causing learning and memory impairment, and did not reverse-regulate these altered RNAs and examine how they affected animal behaviors. Thus, we need to use overexpression or knockdown approaches to regulate the above RNA in future studies to further investigate the mechanism underlying the sarin-induced impairment of cognitive function in the hippocampus.

## 5. Conclusions

In this study, a rat model of repeated low-level sarin exposure was established via the subcutaneous injection of 0.4 × LD_50_ (40 μg/kg) for 21 consecutive days. It was found that rats exposed to sarin had persistent learning and memory dysfunction and a reduced density of the hippocampal dendritic spines. We further studied the molecular mechanism of cognitive impairment caused by sarin exposure using whole-transcriptome sequencing. Compared with the saline control group, a total of 1035 mRNA, 44 miRNA, 305 lncRNA, and 412 circRNA were found to be differentially expressed in the hippocampus of sarin-treated rats. The results of the GO annotation and PPI network revealed that the differential genes were mainly involved in neuronal synaptic plasticity. KEGG enrichment analysis showed that the pathways enriched in these genes were mainly related to neurodegenerative diseases with cognitive defects, such as Alzheimer’s disease and Parkinson’s disease. According to the strict regulatory relationship and interaction between the differential RNAs, the circRNA/lncRNA–miRNA–mRNA ceRNA network was constructed and analyzed. Circ_Fmn1 and Circ_Cacna1c were at the core of the network, in which the up-regulation of Circ_Fmn1 resulted in the down-regulation of miR-741-3p, miR-764-3p, and miR-871-3p, and the up-regulation of the corresponding target mRNAs, such as KIF1A, PTPN11, SYN1, and MT-CO3. Additionally, the down-regulation of Circ_Cacna1c led to the up-regulation of miR-10b-5p and miR-18a-5p, which in turn resulted in the down-regulation of CACNA1C, PRKCD, and RASGRP1. The balance between the two pathways formed by these RNAs is the key to maintaining the stability of synaptic plasticity, which is involved in the process of repeated low-level sarin-induced learning and memory impairment. Our study reveals the ceRNA network that is related to sarin exposure for the first time, suggesting that this particular ceRNA network may be related to the pathogenesis of cognitive impairment induced by repeated low-level sarin exposure. These newly discovered networks reveal potential biomarkers or therapeutic targets for sarin exposure and also offer new insights into the molecular mechanisms of other organophosphorus toxicants.

## Data Availability

The datasets generated during and/or analyzed during the present study are available from the corresponding author on reasonable request.
